# Integration of Cadmium Accumulation, Subcellular Distribution, and Physiological Responses to Understand Cadmium Tolerance in Apple Rootstocks

**DOI:** 10.3389/fpls.2017.00966

**Published:** 2017-06-07

**Authors:** Jiangtao Zhou, Huixue Wan, Jiali He, Deguo Lyu, Huifeng Li

**Affiliations:** ^1^College of Horticulture, Shenyang Agricultural UniversityShenyang, China; ^2^Key Lab of Fruit Quality Development and Regulation of Liaoning ProvinceShenyang, China; ^3^Institute of Pomology, Shandong Academy of Agricultural SciencesTai'an, China

**Keywords:** cadmium (Cd), *Malus* rootstock, subcellular distribution, chemical forms, tolerance

## Abstract

Cadmium (Cd) is a nonessential and highly toxic element causing agricultural problems. However, little information is available about the variation in Cd tolerance among apple rootstocks and its underlying physiological regulation mechanisms. This study investigated Cd accumulation, subcellular distribution, and chemical forms as well as physiological changes among four apple rootstocks exposed to either 0 or 300 μM CdCl_2_. The results showed that variations in Cd tolerance existed among these rootstocks. Cd exposure caused decline in photosynthesis, chlorophyll and biomass in four apple rootstocks, which was less pronounced in *M. baccata*, indicating its higher Cd tolerance. This finding was corroborated with higher Cd tolerance indexes (TIs) of the whole plant in *M. baccata* than those in the other three apple rootstocks. Among the four apple rootstocks, *M. baccata* displayed the lowest Cd concentrations in roots, wood, and leaves, the smallest total Cd amounts as well as the lowest BCF. In apple rootstocks, it was found that to immobilize Cd in cell wall and soluble fraction (most likely in vacuole) and to convert it into pectate- or protein- integrated forms and undissolved Cd phosphate forms may be the primary strategies to reduce Cd mobility and toxicity. The physiological changes including ROS, carbohydrates and antioxidants were in line with the variations of Cd tolerance among four apple rootstocks. In comparison with the other three apple rootstocks, *M. baccata* had lower concentrations of ROS in roots and bark, H_2_O_2_ in roots and leaves and MDA in roots, wood and bark, but higher concentrations of soluble sugars in bark and starch in roots and leaves, and enhanced antioxidants. These results indicate that *M. baccata* are more tolerant to Cd stress than the other three apple rootstocks under the current experiment conditions, which is probably related to Cd accumulation, subcellular partitioning and chemical forms of Cd and well-coordinated antioxidant defense mechanisms.

## Introduction

The application of urban composts, metal-based pesticides, ripening agents, and fertilizers and wastewater irrigation have resulted in increasing cadmium (Cd) accumulation in orchard soil of many parts of the world especially in developing countries (Andreu and Gimeno-García, 1996; Pinamonti et al., 1997; Li et al., 2014; Wang et al., 2015b; Duan et al., 2016). Cd contamination in orchard soil could inhibit net photosynthesis, decrease chlorophyll, repress plant growth, alter carbohydrate concentrations, limit fruit cultivation, and poses a threaten to human health (Hamurcu et al., 2010; López-Climent et al., 2011; Podazza et al., 2012; Stachowiak et al., 2015; Wang et al., 2015b). Clean-up of Cd-contaminated soil is quite difficult and expensive which usually takes several years, or even decades (McGrath and Zhao, 2003). In order to utilize Cd-contaminated soils and minimize the negative effects of Cd to plants or humans, an alternative strategy to reduce the risk of Cd toxicity has been proposed: the selection of crops with low-Cd accumulation and relative high-Cd tolerance by breeding or molecular techniques (Shi and Cai, 2009; Wang et al., 2015a). The huge variations in Cd uptake, accumulation and tolerance among species or cultivars make this choice possible and feasible (Shi and Cai, 2009; López-Climent et al., 2011; Liu et al., 2014). However, this approach requires a mechanistic understanding of Cd toxicity and how it is mobilized within plants at the physiological and genetic levels (Mendoza-Cózatl et al., 2011). Rootstocks of horticultural crops, commonly used for vegetative propagation, not only regulate growth, fertility, and yield, but also affect Cd accumulation, transport and toxicity through ion exclusion or retention (Nawaz et al., 2016; Podazza et al., 2016; Zhou et al., 2016). Previous studies have demonstrated the physiological mechanisms of Cd accumulation, transport, and tolerance in rootstocks of citrus (Podazza et al., 2016), cucumber (Savvas et al., 2013), and pepper (Morikawa, 2017). However, these mechanisms in apple rootstocks remain uncharacterized.

In order to survive in Cd contaminated soil, plants have evolved various strategies for Cd detoxification, including metal exclusion, binding Cd to cell wall, restricting Cd accumulation in sensitive tissues/organelles, sequestration in vacuoles, chelation by organic compounds, and biochemical defenses (Clemens et al., 2013; Luo et al., 2016). In recent years, studies have addressed that subcellular distribution and chemical forms of Cd are associated with Cd accumulation, tolerance and detoxification in plants (Fu et al., 2011; Liu et al., 2014). Subcellular distribution of Cd in plants contains four fractions such as cell wall fraction, organelle-rich fraction, membrane-containing fraction, and soluble fraction (Liu et al., 2014). It was indicated that most plants can compartmentalize Cd in cell wall or soluble fraction to avoid Cd toxicity to organelle or membrane (Wang et al., 2008). Wang et al. (2015a) found that proportions of Cd in cell wall were higher in high-Cd watercress genotypes than in low-Cd groups under Cd stress. Compared to Cd-sensitive barley genotypes, Cd-resistant genotypes accumulated more Cd in cell wall and soluble containing fractions, and less Cd in organelle containing fractions (Wu et al., 2005). Once absorbed by plants, Cd will distribute in different chemical forms, including inorganic, water soluble Cd-organic acid, pectate- and protein-integrated, insoluble phosphate, and oxalate forms (Lai, 2015). To reduce Cd mobility and toxicity in plants, Cd can be converted into undissolved phosphate or pectate- and protein-bound forms (Wu et al., 2005; Wang et al., 2008). However, up to now, the studies have not yet provided consistent results. For example, Fu et al. (2011) found that the greatest amounts of Cd were existed in inorganic forms in *Phytolacca americana* L. The pattern of Cd subcellular partitioning and chemical forms in plants are regarded as key factors affecting the characteristics of Cd migration, accumulation and phytotoxicity degree in different species, cultivars or even in tissues (Wang et al., 2008; Hao et al., 2015). To our knowledge, however, relatively little is known about subcellular distribution and chemical forms of Cd in different apple rootstocks regarding to Cd stress.

In recent years, remarkable progress has been made on elucidating Cd tolerance in crop plants at the physiological levels (Romero-Puertas et al., 2012; Clemens et al., 2013; Zhang et al., 2013; Choppala et al., 2014; Anjum et al., 2016). Cd stress leads to decrease in photosynthesis, chlorophyll contents and thereby resulted in decline in total soluble sugars and starch which are required to provide energy to cope with Cd stress (Nagajyoti et al., 2010; He et al., 2013a). In plants, one of the important reasons for Cd toxicity is the indirect induction of the reactive oxygen species (ROS) including superoxide (${\text{O}}_{2}^{•-}$) and hydrogen peroxide (H_2_O_2_) (Sandalio et al., 2012). The over production of ROS can react with lipids and proteins, which leads to membrane damage (Rodríguez-Serrano et al., 2009). Plants have evolved several protective mechanisms to cope with Cd-induced oxidative stress and reduce its deleterious effects, such as non-enzymatic metabolites including free proline, soluble phenols, and total thiols (T-SH), ascorbate (ASC) and reduced GSH, and antioxidative enzymes including superoxide dismutase (SOD), catalase (CAT), guaiacol peroxidase (GPX), ascorbate peroxidase (APX), and glutathione reductase (GR) (Sun et al., 2013; He et al., 2015; Nahar et al., 2016; Rui et al., 2016). The antioxidative response to Cd exposure varies markedly among plant species (cultivars) and experimental conditions (Xu et al., 2012b). Up to date, limited information is available on the physiological response to Cd stress involved in variations in Cd toxicity and detoxification of different apple rootstocks.

In the present study, to compare Cd accumulation and tolerance among four apple rootstocks, seedlings were exposed to 0 or 300 μM CdCl_2_. The aim of this study is to address the following questions. (1) Are there variations in Cd accumulation and tolerance among different apple rootstocks under high Cd exposure conditions? (2) Are these variations associated with Cd subcellular distribution, chemical forms and physiological regulation mechanisms? To answer the above questions, photosynthesis, chlorophyll, biomass, tolerance index (TI), Cd concentrations, total Cd amounts, bio-concentration factors (BCF), subcellular distribution, and chemical forms of Cd, soluble sugars and starch, oxidants and antioxidants were analyzed. Characterization of mechanisms involved in Cd accumulation and detoxification in apple rootstocks will provide a basis for further screening or engineering Cd-tolerant rootstocks with low Cd accumulation.

## Materials and methods

### Plant cultivation and Cd exposure

Seeds of *Malus baccata* Borkh. (Mb), *M. hupehensis* Rehd. (Mh), *M. micromalus* “qingzhoulinqin” (Mm) and *M. robusta* Rehd. (Mr) were stratified at 0–4°C in sand for 40 days. Subsequently, germinating seeds were cultivated in nursery seedling plate filled seedling matrix. After cultivation for 40 days in a greenhouse under natural light and temperature conditions (day/night temperature, 26/18°C; relative air humidity, 50–60%), uniform seedlings with 6–7 leaves were selected and then transferred to plastic pots (20 × 20 × 18 cm) filled with sand. Each plant was carefully irrigated with 50 ml Hoagland solution in the morning of every 2 days and 100 ml distilled water daily in the evening to avoid runoff. Two weeks later, 24 plants with similar growth performance from each species were divided into two groups (12 plants in each group) and supplied with Hoagland solution containing 0 or 300 μM CdCl_2_, respectively. The Cd treatment lasted for 45 days before harvest.

### Gas exchange measurement and harvest

Before harvest, gas exchange of three mature leaves [leaf plastochron index (LPI) = 7–9] was determined using a CIRAS-2 photosynthesis system (PP Systems, USA). Net photosynthetic rate (*A*), stomatal conductance (*g*_s_), and transpiration rate (*E*) were obtained from each plant per treatment.

After gas exchange measurement, each plant was harvested via separation of root, wood, bark and leaf tissues. The roots were carefully washed to desorb Cd^2+^ from the root surface according to Rauser (1987). Harvested tissues were wrapped with tinfoil after fresh weight of samples were recorded and immediately frozen in liquid nitrogen. Frozen samples were ground into fine powder with a ball mill (Retsch, Haan, Germany) and stored at −80°C. Fresh materials (ca. 50 mg) from each tissue per plant was dried at 60°C for 72 h to determine the fresh-to-dry mass ratio using to calculate the dry weight (biomass) of each tissue.

### Analysis of tolerance index (TI), foliar pigments, Cd, and bio-concentration (BCF)

The tolerance index (TI) was calculated as tissues dry mass of a plant exposed to Cd divided by that under control conditions (Shi and Cai, 2009).

To measure chlorophyll contents in leaves, fine powder of fresh leaves (about 50 mg) was extracted in 5 ml of 80% acetone for 24 h in darkness until the color disappeared completely. The contents of chlorophyll a and chlorophyll b in the extracts were determined spectrophotometrically according to He et al. (2015).

Cd concentrations in different tissues were determined by flame atomic absorbance spectrometry (Hitachi 180-80, Hitachi Ltd, Tokyo, Japan) after digestion with a mixture (7 ml concentrated HNO_3_ and 1 ml concentrated HClO_4_) at 170°C as suggested (He et al., 2013b). The total Cd amounts in each tissue for every plant was calculated by multiplying the Cd concentrations by the dry mass of that tissue. Bio-concentration (BCF) was calculated as metal concentration in plant roots or aerial parts divided by that in the soil or solution (Shi et al., 2010).

### Determination of Cd subcellular distribution and chemical forms

Subcellular distribution of Cd was determined as suggested by Fu et al. (2011). Cells were separated into four fractions: cell wall fraction, organelle-rich fraction, membrane-containing fraction, and soluble fraction using differential centrifugation technique. Briefly, frozen tissues were homogenized in 10 ml of pre-cold (4°C) extraction solution (50 mM HEPES, 500 mM sucrose, 1.0 mM DTT, 5.0 mM ascorbic acid, and 1.0% w:v Polyclar AT PVPP, pH 7.5). The homogenate was sieved through a nylon cloth (100 μm) and the residue was designated as the cell wall fraction (F I) mainly containing cell walls and cell wall debris. The filtrate was centrifuged (10,000 g, 4°C, 30 min) and the pellet containing organelle-rich fraction (F II) was collected. The left supernatant was then centrifuged at 100,000 g, 4°C for 30 min again, and the pellet and supernatant was referred to as the membrane-containing fraction (F III) and soluble fraction (F IV), respectively. All the resultant pellets were re-suspended in extraction buffer. The fractions were dried and wet digested separately, and then Cd concentrations in the digests were determined using flame atomic absorbance spectrometry (Hitachi 180-80, Hitachi Ltd, Tokyo, Japan).

Determination of Cd chemical forms in different apple rootstocks was carried out according to Wu et al. (2005). Cd in different chemical form was extracted in the designated solutions in the following order with the same extraction procedures. (1) 80% ethanol, extracting inorganic Cd including nitrate/nitrite, chloride, and aminophenol cadmium. (2) deionized water, extracting water soluble Cd-organic acid complexes and Cd(H_2_PO_4_)_2_. (3) 1 M NaCl, extracting Cd integrated with pectates and protein. (4) 2% HAC, extracting undissolved Cd phosphate including CdHPO_4_ and Cd_3_(PO_4_)_2_. (5) 0.6 M HCl, extracting cadmium oxalic. Frozen samples were homogenized in the above extraction buffers, diluted at the ratio of 1:100 (w/v). After shaking for 22 h at 25°C, the homogenate was centrifuged (5,000 g, 10 min) and the first supernatant was collected. The pellet was then extracted twice in the same extraction solution and shaked for 2 h at 25°C. After centrifugation (5,000 g, 10 min), the two supernatant was collected and combined with the previous one. Subsequently, the pellet retained in the centrifuge tube was subjected to the next four extraction solution with the same extraction procedures. Each of the pooled supernatant solution was evaporated at 70°C to constant weight, and digested with HNO_3_ at 145°C and then Cd concentrations were determined by flame atomic absorbance spectrometry (Hitachi 180-80, Hitachi Ltd, Tokyo, Japan).

### Determination of ${\text{O}}_{2}^{•-}$, H_2_O_2_ and MDA

The concentrations of ${\text{O}}_{2}^{•-}$ and H_2_O_2_ in root, wood, bark and leaf tissues were measured using a spectrophotometer at 530 and 410 nm, respectively, according to the method of He et al. (2015).

The concentrations of malonaldehyde (MDA) in samples were determined spectrophotometrically at 450, 532, and 600 nm as suggested by He et al. (2015).

### Analysis of total soluble sugars and starch

The concentrations of total soluble sugars and starch were determined using the anthrone method as suggested (Yemm and Willis, 1954). The fine powder (about 100 mg) were extracted in 80% ethanol for 30 min at 80°C and then centrifuged at 6,000 g for 10 min. After the collection of the first supernatant, the pellet was extracted again as mentioned above and the two supernatant was combined together. The absorbance of the mixture was recorded at 620 nm spectrophotometrically after adding anthrone reagent to the supernatant and heating in boiling water. The standard curve was generated by a serial of diluted glucose solutions.

To analyze starch in different tissues, the pellet retained after the extraction of the soluble sugars was further extracted by HClO_4_. Starch in the supernatant was determined as above.

### Analysis of non-enzymatic metabolites and antioxidative enzyme activities

The concentrations of non-enzymatic metabolites including free proline, soluble phenols, and total thiols (T-SH) were analyzed spectrophotometrically as reported by He et al. (2013b). Ascorbate (ASC) and reduced GSH were determined by the procedure described by Chen et al. (2011).

The soluble proteins were extracted from fresh materials and quantified according to Luo et al. (2008). The activity of superoxide dismutase (SOD), catalase (CAT), guaiacol peroxidase (GPX), ascorbate peroxidase (APX) were determined as described by He et al. (2011), and glutathione reductase (GR) according to Wang et al. (2013).

### Statistical analysis

All statistical tests were performed by Statgraphics (STN, St Louis, MO, USA). Before statistical analysis, all data were tested for normality. For all parameters, two-way analysis of variance (ANOVAS) were applied, with CdCl_2_ (Cd) and rootstock (R) as two main factors. A posteriori comparison of means was done when the interaction was significant. In order to reduce the chance of type I errors, all *P*-values obtained from multiple comparisons were corrected by Tukey-HSD method. Differences between means were considered significant when the *P*-value of the ANOVA F-test was < 0.05.

## Results

### Plant growth and tolerance index

After 300 μM Cd exposure for 45 days, CO_2_ assimilation rate (*A*) of mature leaves were repressed by 26.6, 36.9, 35.3, and 21.4%, respectively, in *M. baccata, M. hupehensis, M. micromalus* “qingzhoulinqin” and *M. robusta* compared to those in control plants (Table S1). However, except for stomatal conductance (*g*_s_) in *M. hupehensis, g*_*s*_ and transpiration rate (*E*) in all of other rootstocks were not affected by Cd exposure (Table S1). Concentrations of chlorophyll a were markedly reduced by 6.7% in *M. hupehensis*, but were unaffected in other three apple rootstocks exposed to Cd compared to those without Cd exposure. Treatment with 300 μM Cd markedly reduced the concentrations of chlorophyll b and chlorophyll (a + b) in all plants in comparison with those in controls (Table S1).

In order to assess the toxic effects of Cd on plant growth, biomass of different rootstocks were analyzed (Table S2). In four apple rootstocks, the wood, bark and leaf biomass were negatively affected by Cd exposure. The reduction in total biomass was smallest in *M. baccata* (7.0%), followed by *M. micromalus* “qingzhoulinqin” (13.7%), *M. robusta* (13.7%), and *M. hupehensis* (21.1%) (Table S2).

Tolerance indexes (TIs) which could reflect the ability of plant tolerance to Cd in four apple rootstocks are shown in Table S3. The TIs of root and wood did not differ among four apple rootstocks, whereas TIs of bark and leaf exhibited a clear species variation after Cd exposure. The TIs of the whole plant in *M. baccata* was 7.7–17.8% higher than those of the other three apple rootstocks (Table S3).

### Cd concentrations, total Cd and BCF

**Figure 1:**
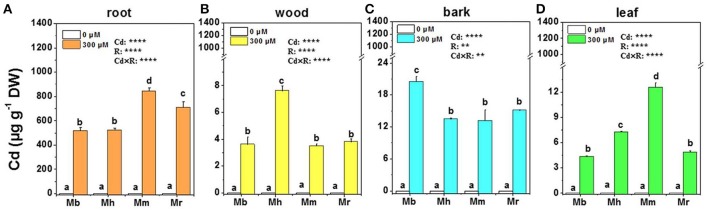
Cd concentrations in root **(A)**, wood **(B)**, bark **(C)**, and leaf **(D)** tissues of four apple rootstocks exposed to 0 or 300 μM CdCl_2_ for 45 days. Bars indicate means ± SE (*n* = 6). Different letters on the bars for the same tissue indicate significant difference between the treatments. *P*-values of the ANOVAs of Cd^2+^ (Cd), rootstocks (R), and their interaction (Cd×R) are indicated. ^**^P ≤ 0.01; ^****^P ≤ 0.0001; ns, not significant. Mb, *Malus. baccata* Borkh.; Mh, *M. hupehensis* Rehd.; Mm, *M. micromalus* “qingzhoulinqin”; Mr, *M. robusta* Rehd.

Treatment with 300 μM Cd caused Cd accumulation in roots, wood, bark and leaves of all plants compared to that in the controls without Cd exposure, and the changes were species-specific (Figure 1). Generally, Cd concentrations in different tissues of all apple rootstocks were highest in roots, followed by bark, leaves and wood. Among four apple rootstocks, *M. baccata* displayed the lowest Cd concentrations in all tissues of Cd exposed plants except for bark (Figure 1). In roots and leaves, Cd concentrations were highest in *M. micromalus* “qingzhoulinqin,” which exhibited 18.7–63.0%, 73.5–191.3% higher than those in other three apple rootstocks, respectively (Figures 1A,D).

**Figure 2:**
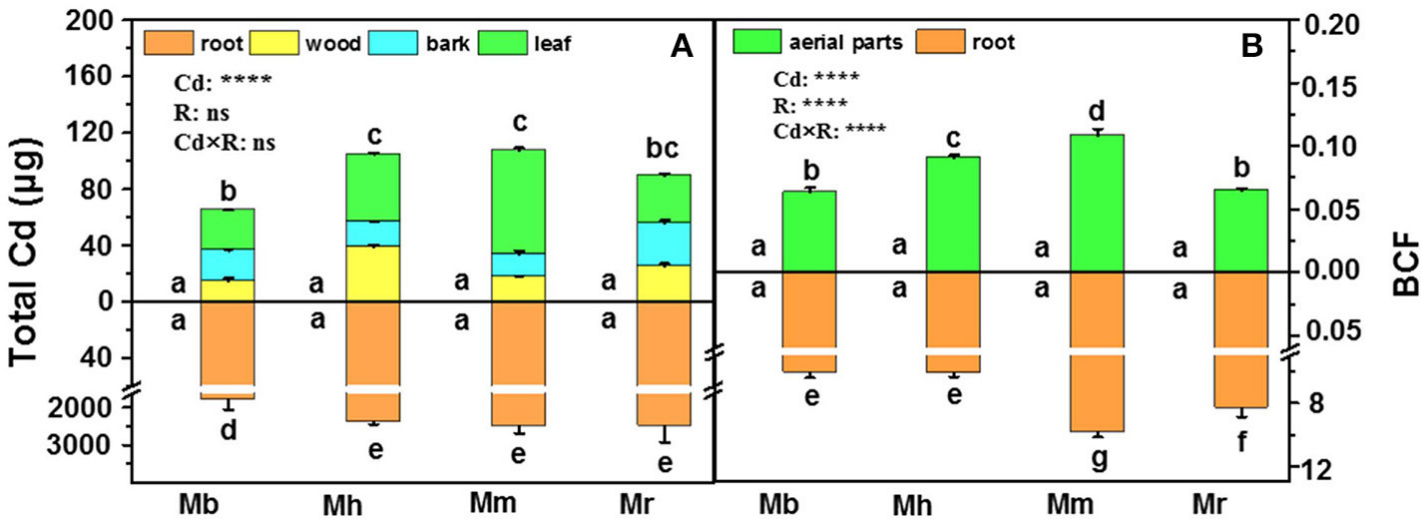
Total Cd amounts **(A)** in root, wood, bark and leaf tissues and bio-concentration factor (BCF, B) in root and aerial parts of four apple rootstocks exposed to 0 or 300 μM CdCl_2_ for 45 days. Bars indicate means ± SE (*n* = 6). Different letters on the bars for the same tissue indicate significant difference between the treatments. *P*-values of the ANOVAs of Cd^2+^ (Cd), rootstocks (R), and their interaction (Cd×R) are indicated. ^****^P ≤ 0.0001; ns, not significant. Mb, *Malus. baccata* Borkh.; Mh, *M. hupehensis* Rehd.; Mm, *M. micromalus* “qingzhoulinqin”; Mr, *M. robusta* Rehd.

In this study, total Cd amounts in all plants were calculated on the basis of Cd concentrations in tissues and their corresponding biomass (Figure 2A). Total Cd amounts in roots and the aerial parts were similar in *M. hupehensis, M. micromalus* “qingzhoulinqin” and *M. robusta*, whereas significantly lower total Cd amounts in these tissues were found in *M. baccata* compared with the other three apple rootstocks after Cd exposure (Figure 2A), mainly due to its relative low Cd concentrations. In order to evaluate the ability of plants to accumulate Cd, BCFs of different apple rootstocks were calculated (Figure 2B). Among the four apple rootstocks, root BCFs were higher than aerial parts BCFs. For both roots and aerial parts, the lowest and highest BCFs were found in *M. baccata* and *M. micromalus* “qingzhoulinqin,” respectively (Figure 2B).

### Subcellular distribution of Cd

After 300 μM Cd exposure for 45 days, there was a pronounced difference in subcellular distribution of Cd in the different fractions of four apple rootstocks (Table 1). In general, Cd mainly stored in cell wall fraction (F I), followed by soluble fraction (F IV), organelle-rich fraction (F II), and membrane-containing fraction (F III) in root, bark and leaf tissues of different apple rootstocks. However, in wood, Cd subcellular distribution of four apple rootstocks decreased in the order F IV > F I > F II > F III (Table 1).

**Table 1:** Subcellular distribution of Cd and its proportion in root, wood, bark and leaf tissues of four apple rootstocks exposed to 300 μM CdCl_2_ for 45 days.

**Tissues**	**Rootstock**	**Cd concentration (μg g**^**−1**^ **DW)**				**Cd distribution ratio (%)**			
		**F I**	**F II**	**F III**	**F IV**	**F I**	**F II**	**F III**	**F IV**
Root	Mb	254.35 ± 4.07 b	92.11 ± 7.24 a	15.58 ± 0.45 a	129.41 ± 3.03 a	51.76 ± 1.01 c	18.74 ± 1.42 a	3.17 ± 0.08 a	26.33 ± 0.56 a
	Mh	230.67 ± 3.98 ab	115.07 ± 3.80 ab	18.24 ± 0.03 a	131.17 ± 0.51 a	46.58 ± 0.64 c	23.24 ± 0.73 ab	3.68 ± 0.03 b	26.49 ± 0.25 a
	Mm	237.06 ± 11.11 b	137.27 ± 15.61 b	25.74 ± 2.45 b	194.39 ± 14.03 b	39.92 ± 2.05 a	23.11 ± 2.57 ab	4.52 ± 0.24 c	30.99 ± 0.57 b
	Mr	207.35 ± 12.70 a	136.62 ± 2.14 b	16.87 ± 0.36 a	142.67 ± 12.14 a	41.20 ± 2.61 ab	27.14 ± 0.52 b	3.35 ± 0.06 ab	28.31 ± 2.26 ab
	*P*-value	^*^	^*^	^***^	^**^	^**^	^*^	^***^	ns
Wood	Mb	1.64 ± 0.06 b	0.44 ± 0.03 a	0.11 ± 0.00 a	1.64 ± 0.13 a	43.00 ± 1.03 b	11.35 ± 0.19 b	2.93 ± 0.08 a	42.71 ± 0.93 a
	Mh	2.41 ± 0.01 c	0.60 ± 0.06 b	0.24 ± 0.00 c	3.94 ± 0.11 b	33.52 ± 0.69 a	8.32 ± 0.67 a	3.38 ± 0.06 b	54.78 ± 0.56 b
	Mm	0.85 ± 0.08 a	0.42 ± 0.03 a	0.10 ± 0.00 a	1.78 ± 0.10 a	26.99 ± 2.93 a	13.38 ± 0.82 bc	3.29 ± 0.06 b	56.34 ± 2.06 b
	Mr	0.91 ± 0.03 a	0.47 ± 0.03 ab	0.14 ± 0.01 b	1.61 ± 0.21 a	29.41 ± 3.07 a	15.10 ± 0.64 c	4.40 ± 0.06 c	51.09 ± 3.36 b
	*P*-value	^****^	^*^	^****^	^****^	^**^	^***^	^****^	^**^
Bark	Mb	8.72 ± 0.26 c	4.95 ± 0.22 b	0.19 ± 0.01 b	4.84 ± 0.28 bc	46.69 ± 1.72 b	26.44 ± 0.85 b	1.01 ± 0.09 a	25.86 ± 1.35 a
	Mh	4.61 ± 0.04 a	3.09 ± 0.32 a	0.23 ± 0.00 c	5.97 ± 0.32 c	33.17 ± 0.25 a	22.22 ± 2.35 ab	1.67 ± 0.03 b	42.94 ± 2.24 b
	Mm	6.56 ± 0.31 b	4.48 ± 0.28 b	0.14 ± 0.01 a	2.91 ± 0.30 a	46.65 ± 2.16 b	31.74 ± 0.68 c	0.99 ± 0.04 a	20.63 ± 1.88 a
	Mr	8.81 ± 0.18 c	3.30 ± 0.08 a	0.15 ± 0.02 ab	3.70 ± 0.70 ab	55.39 ± 2.74 c	20.76 ± 1.08 a	0.95 ± 0.12 a	22.90 ± 3.57 a
	*P*-value	^****^	^**^	^**^	^**^	^***^	^**^	^***^	^***^
Leaf	Mb	2.69 ± 0.11 a	0.54 ± 0.03 a	0.09 ± 0.00 a	1.47 ± 0.07 a	56.16 ± 0.82 c	11.27 ± 0.83 a	1.88 ± 0.06 a	30.72 ± 0.07 a
	Mh	2.80 ± 0.13 b	1.37 ± 0.13 b	0.12 ± 0.01 a	2.31 ± 0.15 a	42.54 ± 2.63 b	20.73 ± 1.27 b	1.88 ± 0.13 a	34.85 ± 1.26 a
	Mm	5.37 ± 0.25 c	2.35 ± 0.06 c	0.31 ± 0.04 b	4.00 ± 0.45 b	44.67 ± 1.15 b	19.66 ± 1.15 b	2.61 ± 0.20 b	33.06 ± 2.06 a
	Mr	1.28 ± 0.06 a	0.78 ± 0.11 a	0.11 ± 0.01 a	1.99 ± 0.39 a	31.64 ± 3.20 a	18.68 ± 0.27 b	2.73 ± 0.14 b	46.95 ± 3.10 b
	*P*-value	^****^	^****^	^***^	^**^	^**^	^****^	^**^	^**^

*Data indicate means ± SE (n = 6). Different letters behind the values in the same column indicate significant difference between the treatments. P-values of the ANOVAs of rootstock were also shown*.

* P ≤ 0.05;

** P ≤ 0.01;

*** P ≤ 0.001;

**** *P ≤ 0.0001; ns, not significant. F I, cell wall fraction; F II, organelle-rich fraction; F III, membrane-containing fraction; F IV, soluble fraction. Mb, M. baccata Borkh.; Mh, M. hupehensis Rehd.; Mm, M. micromalus “qingzhoulinqin”; Mr, M. robusta Rehd*.

The subcellular distribution of Cd in different fractions of tissues differed among four apple rootstocks (Table 1). In roots, Cd concentrations and distribution ratios of F I were highest in *M. baccata*, while the highest Cd concentrations and proportions of F IV were found in *M. micromalus* “qingzhoulinqin.” In comparison with other three apple rootstocks, the lowest Cd concentrations and proportions of F II and F III in roots were observed in *M. baccata* (Table 1). Cd distribution ratio of F I in wood of *M. baccata* was higher than that of other three apple rootstocks, whereas the opposite pattern applied to Cd distribution ratio of F IV. Among four apple rootstocks, Cd proportions of F II and F III in wood of *M. baccata* and *M. hupehensis* were relative lower than those of *M. micromalus* “qingzhoulinqin” and *M. robusta*. Cd concentrations of F I and F IV in bark were highest in *M. baccata* and *M. robusta*, and *M. baccata* and *M. hupehensis*, respectively, whereas Cd concentrations of F III were highest in *M. hupehensis* (Table 1). In leaves, the highest Cd distribution ratio of F I, and the lowest Cd concentrations and distribution ratio of F II and F III were all found in *M. baccata* (Table 1).

### Chemical forms of Cd

Chemical forms of Cd is closely connected with its biological function, and different Cd chemical forms have distinct toxicity to plant cells and migration capabilities (Fu et al., 2011). To explore the mechanisms regarding Cd translocation and detoxification, chemical forms of Cd in tissues of four apple rootstocks were examined (Table 2). Among four apple rootstocks, Cd concentrations of all chemical forms were always highest in roots, followed by bark, leaves, and wood (Table 2), corresponding well with Cd concentrations in tissues (Figure 1). It was noticed that Cd concentrations and proportion in pectates and protein integrated forms (extracted by 1 M NaCl) were highest, followed by water soluble fraction (extracted by deionized water and corresponded to the most toxic form of metals) in root, wood, and bark tissues, irrespective of apple rootstocks species (Table 2). However, in leaves, the Cd forms extracted by 1 M NaCl and 2% HAC (undissolved Cd phosphate form) were predominant, representing 31.1 and 34.4% of all chemical forms, respectively, averaged over four apple rootstocks. In general, Cd concentrations and proportions of inorganic form (extracted by 80% ethanol), cadmium oxalate form (extracted by 0.6 M HCl) in all tissues, and undissolved Cd phosphate form in all tissues except in leaves were very low in apple rootstocks (Table 2).

**Table 2:** Different chemical forms of Cd and its proportion in root, wood, bark and leaf tissues of four apple rootstocks exposed to 300 μM CdCl_2_ for 45 days.

**Tissues**	**Rootstock**	**Cd concentrations (μg g**^**−1**^ **DW)**					**Percentage of Cd in Different chemical forms (%)**				
		**80% ethanol**	**d-H_2_O**	**1 M NaCl**	**2% HAC**	**0.6 M HCl**	**80% ethanol**	**d-H_2_O**	**1 M NaCl**	**2% HAC**	**0.6 M HCl**
Root	Mb	19.25 ± 0.72 a	130.20 ± 12.52 a	303.24 ± 3.47 a	29.63 ± 0.43 a	1.26 ± 0.08 a	4.00 ± 0.01 ab	26.58 ± 1.68 a	62.78 ± 1.42 b	6.14 ± 0.27 a	0.26 ± 0.02 b
	Mh	19.76 ± 0.16 a	125.35 ± 6.84 a	313.46 ± 5.14 a	51.73 ± 3.83 b	1.25 ± 0.05 a	3.86 ± 0.03 a	24.51 ± 1.44 a	61.25 ± 0.74 b	10.10 ± 0.71 c	0.24 ± 0.01 b
	Mm	33.57 ± 2.23 b	318.14 ± 11.51 c	416.59 ± 8.08 b	68.43 ± 3.71 c	1.18 ± 0.12 a	4.00 ± 0.24 ab	37.94 ± 1.04 b	49.73 ± 1.41 a	8.16 ± 0.39 b	0.14 ± 0.01 a
	Mr	31.98 ± 1.49 b	257.57 ± 15.94 b	330.62 ± 20.66 a	85.92 ± 4.40 d	2.71 ± 0.13 b	4.52 ± 0.28 b	36.34 ± 2.02 b	46.61 ± 2.44 a	12.12 ± 0.56 d	0.38 ± 0.02 c
	*P*-value	^****^	^****^	^***^	^****^	^****^	*ns*	^***^	^***^	^***^	^****^
Wood	Mb	0.08 ± 0.00 a	0.73 ± 0.03 a	2.48 ± 0.04 b	0.04 ± 0.01 a	0.02 ± 0.00 b	2.39 ± 0.07 a	21.65 ± 0.81 a	73.50 ± 0.83 c	1.27 ± 0.13 ab	0.65 ± 0.10 b
	Mh	0.21 ± 0.02 b	1.62 ± 0.02 c	5.06 ± 0.09 c	0.12 ± 0.00 b	0.02 ± 0.00 b	2.94 ± 0.26 ab	23.06 ± 0.19 ab	71.93 ± 0.41 c	1.64 ± 0.02 b	0.35 ± 0.03 a
	Mm	0.42 ± 0.05 c	0.90 ± 0.04 b	1.63 ± 0.08 a	0.03 ± 0.01 a	0.04 ± 0.00 c	13.92 ± 1.63 c	29.63 ± 1.66 c	53.85 ± 2.04 a	1.15 ± 0.23 a	1.20 ± 0.06 c
	Mr	0.20 ± 0.01 b	0.89 ± 0.01 b	2.40 ± 0.00 b	0.04 ± 0.00 a	0.01 ± 0.00 a	5.50 ± 0.21 b	25.19 ± 0.11 b	67.52 ± 0.34 b	1.08 ± 0.04 a	0.40 ± 0.00 a
	*P*-value	^****^	^****^	^****^	^****^	^**^	^****^	^**^	^****^	ns	^****^
Bark	Mb	0.98 ± 0.05 c	3.97 ± 0.02 c	14.25 ± 0.11 d	0.46 ± 0.02 c	0.45 ± 0.02 c	4.87 ± 0.22 c	19.66 ± 0.04 a	70.60 ± 0.10 ab	2.26 ± 0.12 c	2.23 ± 0.12 b
	Mh	0.46 ± 0.06 b	3.08 ± 0.14 b	8.61 ± 0.09 a	0.22 ± 0.02 b	0.28 ± 0.01 b	3.63 ± 0.45 b	24.32 ± 0.94 b	68.03 ± 1.20 a	1.75 ± 0.19 b	2.18 ± 0.03 b
	Mm	0.44 ± 0.02 ab	2.59 ± 0.19 a	9.76 ± 0.23 b	0.05 ± 0.00 a	0.21 ± 0.01 a	3.37 ± 0.16 b	19.81 ± 1.39 a	74.66 ± 1.43 b	0.39 ± 0.03 a	1.57 ± 0.04 a
	Mr	0.32 ± 0.03 a	3.03 ± 0.07 b	11.16 ± 0.29 c	0.06 ± 0.00 a	0.26 ± 0.01 b	2.12 ± 0.21 a	20.34 ± 0.69 a	74.87 ± 0.84 b	0.43 ± 0.02 a	1.75 ± 0.07 a
	*P*-value	^****^	^***^	^****^	^****^	^****^	^***^	^*^	^**^	^****^	^***^
Leaf	Mb	0.15 ± 0.00 a	0.52 ± 0.03 a	0.51 ± 0.02 a	2.32 ± 0.09 b	0.41 ± 0.01 b	3.94 ± 0.11 a	13.37 ± 0.65 a	13.04 ± 0.77 a	59.03 ± 1.08 d	10.39 ± 0.57 c
	Mh	0.38 ± 0.03 b	0.69 ± 0.01 a	3.18 ± 0.42 b	2.45 ± 0.04 b	0.52 ± 0.04 c	5.56 ± 0.35 b	10.11 ± 0.33 a	45.48 ± 3.35 b	35.56 ± 1.98 c	7.52 ± 0.89 b
	Mm	0.44 ± 0.02 b	2.95 ± 0.08 b	6.09 ± 0.34 c	2.38 ± 0.01 b	0.77 ± 0.04 d	3.48 ± 0.16 a	23.40 ± 0.91 c	48.13 ± 1.64 b	18.85 ± 0.48 a	6.08 ± 0.26 b
	Mr	0.47 ± 0.08 b	2.72 ± 0.19 b	1.06 ± 0.18 a	1.42 ± 0.12 a	0.24 ± 0.02 a	7.89 ± 0.61 c	46.13 ± 1.27 d	17.77 ± 1.49 a	23.97 ± 0.25 b	4.15 ± 0.17 a
	*P*-value	^**^	^****^	^****^	^****^	^****^	^****^	^****^	^****^	^****^	^***^

*Data indicate means ± SE (n = 6). Different letters behind the values in the same column indicate significant difference between the treatments. P-values of the ANOVAs of rootstocks were also shown*.

* P ≤ 0.05;

** P ≤ 0.01;

*** P ≤ 0.001;

**** *P ≤ 0.0001; ns, not significant. Mb, M. baccata Borkh.; Mh, M. hupehensis Rehd.; Mm, M. micromalus “qingzhoulinqin”; Mr, M. robusta Rehd*.

The concentrations and proportions of different Cd chemical forms varied markedly with respect to four apple rootstocks (Table 2). In roots, both concentrations and proportions of inorganic and organic water-soluble Cd, with higher capacity to migrate and higher toxicity to plant cells, were lower in *M. baccata* and *M. hupehensis* than in *M. micromalus* “qingzhoulinqin” and *M. robusta*. However, in roots of *M. baccata*, the proportions of Cd in pectates and protein integrated form were 26.2–34.7% higher than those in *M. micromalus* “qingzhoulinqin” and *M. robusta*, and the proportions of Cd in cadmium oxalate form were 85.7% higher than those in *M. micromalus* “qingzhoulinqin,” respectively (Table 2). Similar results of Cd concentrations and proportions in different chemical forms were also observed for wood among four apple rootstocks (Table 2). In bark, the highest Cd concentrations of five chemical forms were all found in *M. baccata* in comparison with those of other three apple rootstocks, which may be attributed to its relative higher Cd concentrations in bark. Similarly, Cd proportions in inorganic form, undissolved Cd phosphate form and cadmium oxalate form were higher by 34.2–129.7, 29.1–479.5, and 2.3–42.0% in bark of *M. baccata*, compared to those in the other three apple rootstocks (Table 2). In comparison of the other three apple rootstocks, leaves of *M. baccata* displayed the relative lower Cd concentrations and proportions in the first three chemical forms, whereas the highest Cd proportions in the last two chemical forms (Table 2).

### ${\text{O}}_{2}^{•-}$, H_2_O_2_, and MDA

**Figure 3:**
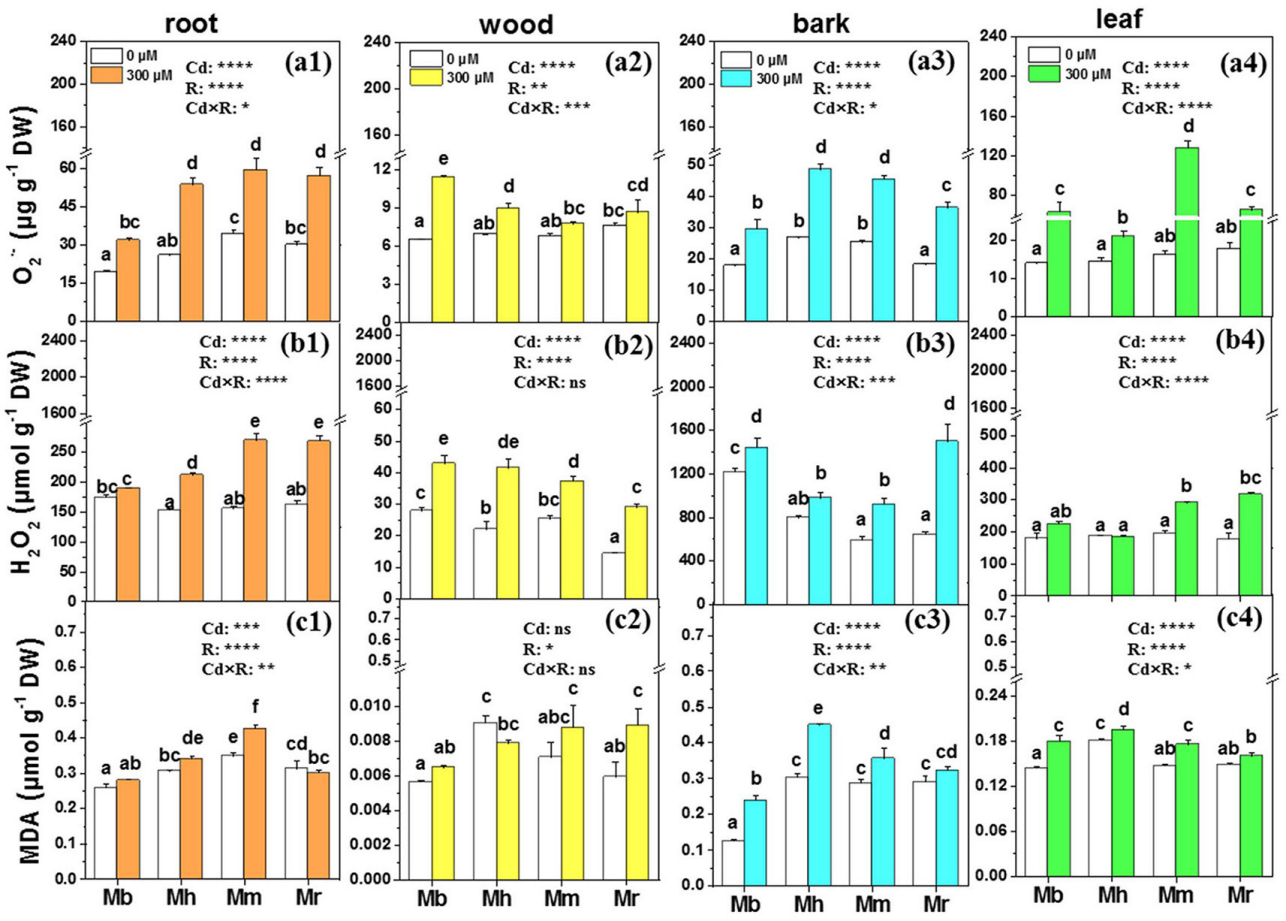
${\text{O}}_{2}^{•-}$, H_2_O_2_ and MDA in root **(a1,b1,c1)**, wood **(a2,b2,c2)**, bark **(a3,b3,c3)**, and leaf **(a4,b4,c4)** tissues of four apple rootstocks exposed to 0 or 300 μM CdCl_2_ for 45 days. Bars indicate means ± SE (*n* = 6). Different letters on the bars for the same tissue indicate significant difference between the treatments. *P*-values of the ANOVAs of Cd^2+^ (Cd), rootstocks (R), and their interaction (Cd×R) are indicated. ^*^P ≤ 0.05; ^**^P ≤ 0.01; ^***^P ≤ 0.001; ^****^P ≤ 0.0001; ns, not significant. Mb, *Malus. baccata* Borkh.; Mh, *M. hupehensis* Rehd.; Mm, *M. micromalus* “qingzhoulinqin”; Mr, *M. robusta* Rehd.

${\text{O}}_{2}^{•-}$ and H_2_O_2_, induced and imbalanced in plants after Cd exposure, were measured in the present study to assess Cd toxicity in apple rootstocks (Figure 3). Concentrations of ${\text{O}}_{2}^{•-}$ were remarkably enhanced by 64.4, 106.9, 73.2, and 90.2% in roots of *M. baccata, M. hupehensis, M. micromalus* “qingzhoulinqin” and *M. robusta*, respectively, under Cd treatment compared to those under control conditions (Figure 3). Similarly, Cd exposure led to significant increases in ${\text{O}}_{2}^{•-}$ accumulation in bark and leaves of four apple rootstocks, but this effect was less pronounced in *M. baccata* (Figure 3). H_2_O_2_ concentrations were significantly enhanced in all tissues of plants with the exception of roots of *M. baccata* and leaves of *M. baccata* and *M. hupehensis*, under Cd addition in comparison with those under controls (Figure 3).

MDA which is an indicator for membrane lipid oxidation was also determined in four apple rootstocks to evaluate the ability of plants to tolerate Cd stress (Figure 3). Changes in MDA concentrations were species-specific. MDA concentrations in roots, bark and leaves of *M. baccata* were lowest compared to the other three apple rootstocks (Figure 3). No effects of Cd exposure on MDA concentrations were found in roots and wood of *M. baccata*. Cd exposure did not affect MDA concentrations in bark and leaves of *M. robusta*, but led to significant increases in these tissues in the other three apple rootstocks.

### Total soluble sugars and starch

**Figure 4:**
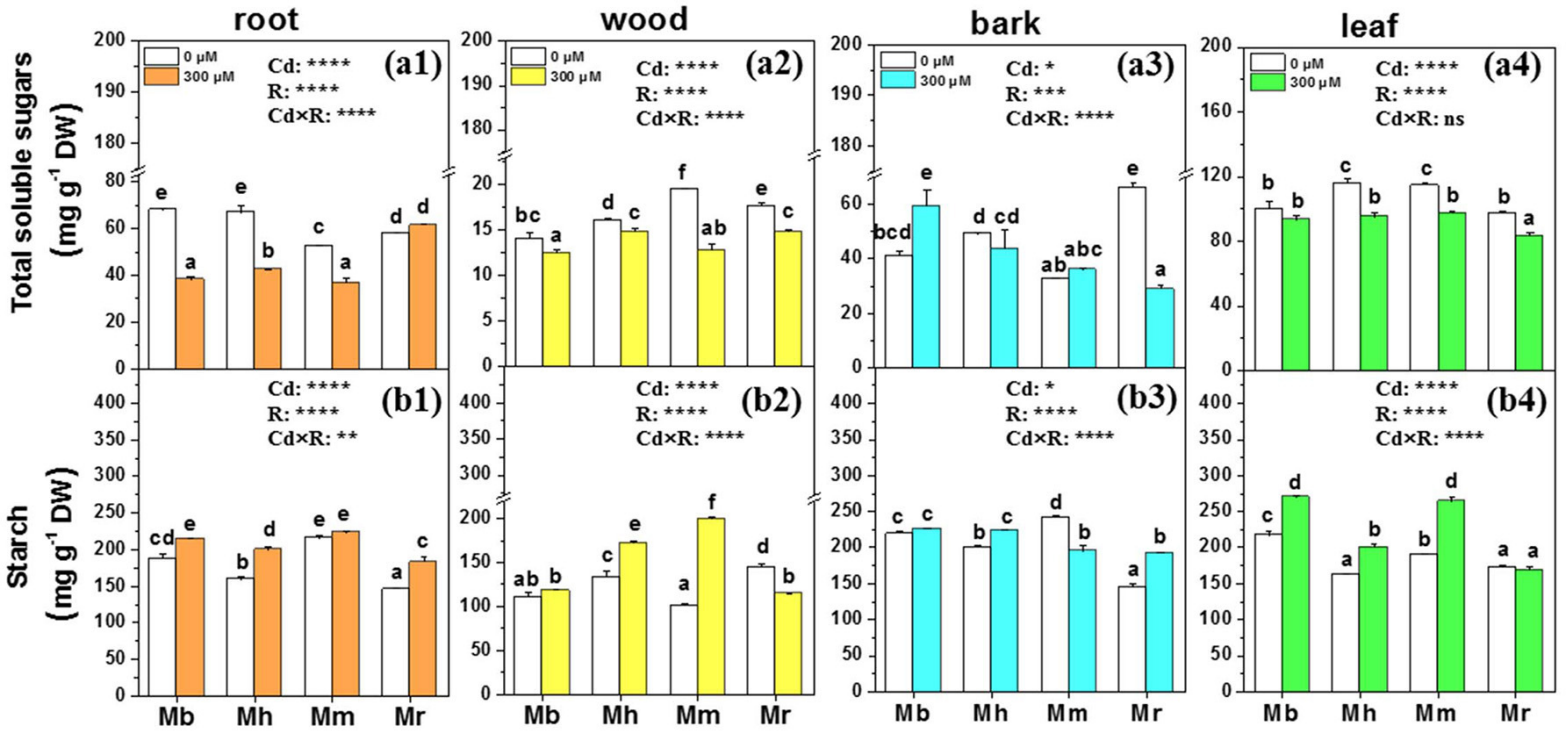
Total soluble sugars and starch in root **(a1,b1)**, wood **(a2,b2)**, bark **(a3,b3)**, and leaf **(a4,b4)** tissues of four apple rootstocks exposed to 0 or 300 μM CdCl_2_ for 45 days. Bars indicate means ± SE (*n* = 6). Different letters on the bars for the same tissue indicate significant difference between the treatments. *P*-values of the ANOVAs of Cd^2+^ (Cd), rootstocks (R), and their interaction (Cd×R) are indicated. ^*^P ≤ 0.05; ^**^P ≤ 0.01; ^***^P ≤ 0.001; ^****^P ≤ 0.0001; ns, not significant. Mb, *Malus. baccata* Borkh.; Mh, *M. hupehensis* Rehd.; Mm, *M. micromalus* “qingzhoulinqin”; Mr, *M. robusta* Rehd.

Total soluble sugars and starch are important metabolites and play vital roles in osmotic regulation, membrane lipid biosynthesis and detoxification of oxidants under Cd treatment conditions (He et al., 2015). Therefore, concentrations of total soluble sugars and starch in tissues of apple rootstocks were determined (Figure 4). The effects of Cd stress on total soluble sugars and starch were species-specific. Cd treatment decreased total soluble sugars in roots, wood and leaves of all plants except for roots of *M. robusta* and leaves of *M. baccata*, compared to control plants (Figure 4). Among the four apple rootstocks, only *M. baccata* displayed an increase of total soluble sugars in bark (Figure 4). Generally, the mean concentrations of starch were enhanced in root, wood, bark, and leaf tissues of apple rootstocks exposed to Cd compared to those under control conditions (Figure 4). Among the four apple rootstocks, the concentrations of starch were significantly higher in roots, bark and leaves of *M. baccata* and *M. micromalus* “qingzhoulinqin” than in these tissues of *M. hupehensis* and *M. robusta* (Figure 4).

### Non-enzymatic and enzymatic antioxidants

**Figure 5:**
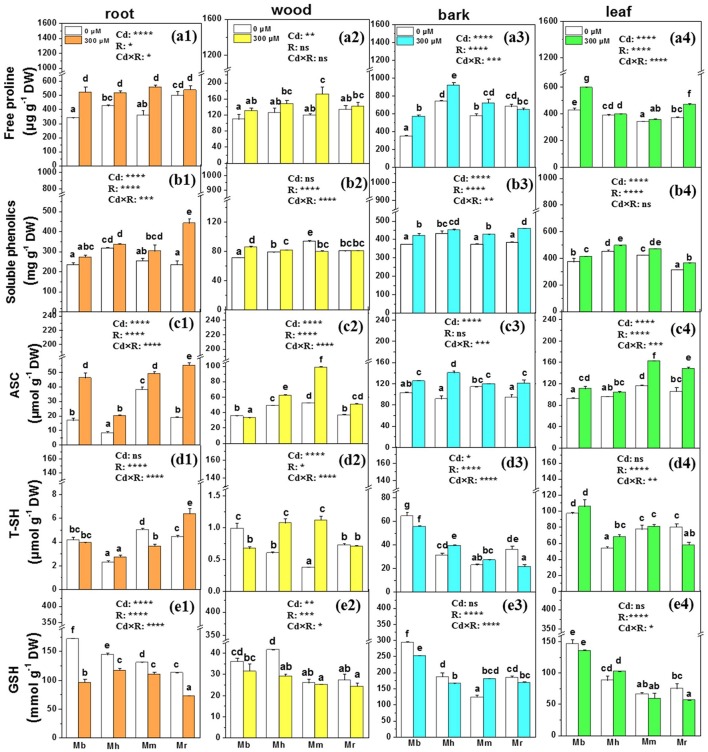
Free proline, soluble phenolics, ASC, T-SH, and GSH in root **(a1,b1,c1,d1,e1)**, wood **(a2,b2,c2,d2,e2)**, bark **(a3,b3,c3,d3,e3)**, and leaf **(a4,b4,c4,d4,e4)** tissues of four apple rootstocks exposed to 0 or 300 μM CdCl_2_ for 45 days. Bars indicate means ± SE (*n* = 6). Different letters on the bars for the same tissue indicate significant difference between the treatments. *P*-values of the ANOVAs of Cd^2+^ (Cd), rootstocks (R), and their interaction (Cd×R) are indicated. ^*^P ≤ 0.05; ^**^P ≤ 0.01; ^***^P ≤ 0.001; ^****^P ≤ 0.0001; ns, not significant. Mb, *Malus. baccata* Borkh.; Mh, *M. hupehensis* Rehd.; Mm, *M. micromalus* “qingzhoulinqin”; Mr, *M. robusta* Rehd.

**Figure 6:**
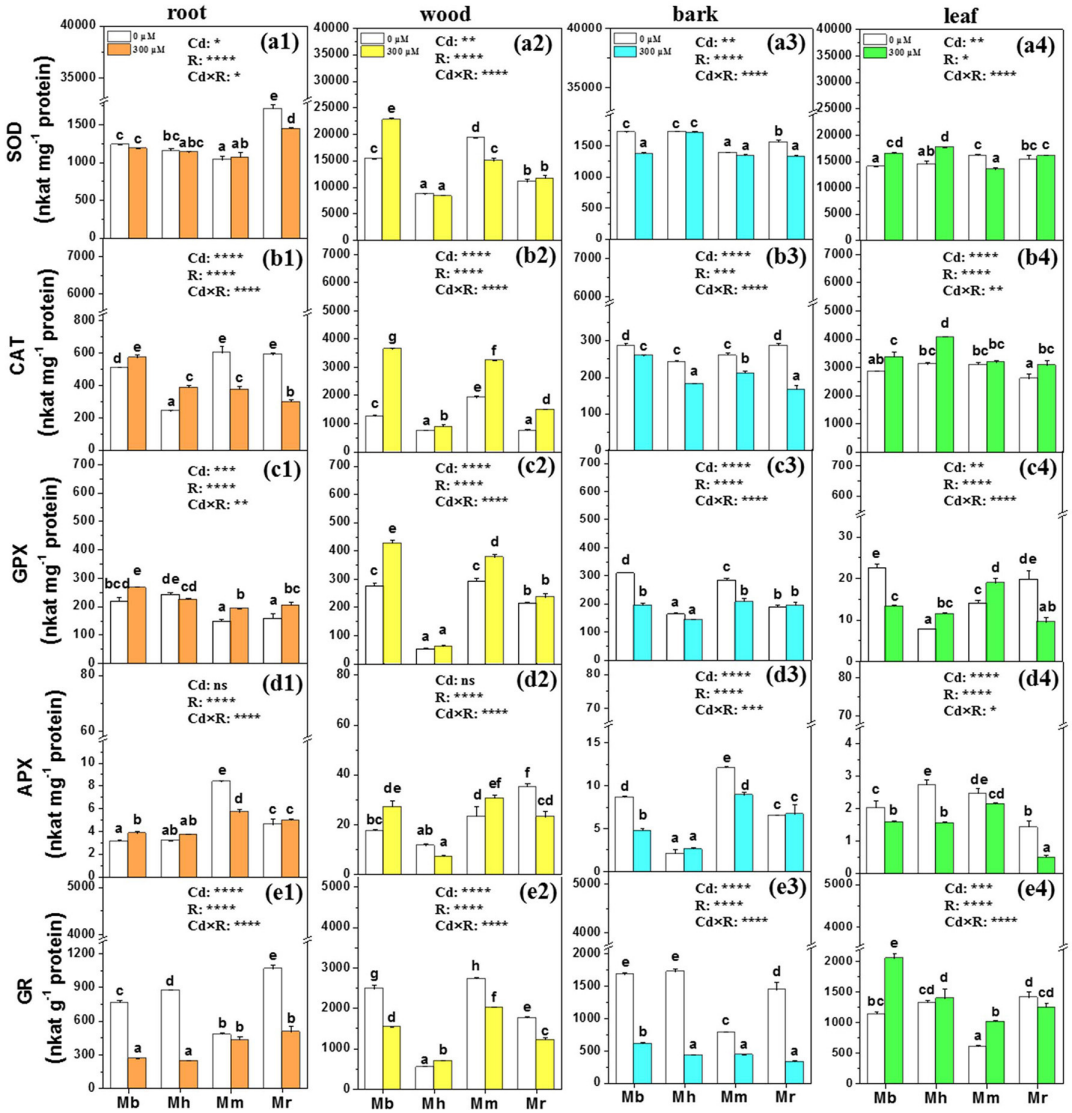
SOD, CAT, GPX, APX, and GR in root **(a1,b1,c1,d1,e1)**, wood **(a2,b2,c2,d2,e2)**, bark **(a3,b3,c3,d3,e3)**, and leaf **(a4,b4,c4,d4,e4)** tissues of four apple rootstocks exposed to 0 or 300 μM CdCl_2_ for 45 days. Bars indicate means ± SE (*n* = 6). Different letters on the bars for the same tissue indicate significant difference between the treatments. *P*-values of the ANOVAs of Cd^2+^ (Cd), rootstocks (R), and their interaction (Cd×R) are indicated. ^*^P ≤ 0.05; ^**^P ≤ 0.01; ^***^P ≤ 0.001; ^****^P ≤ 0.0001; ns, not significant. Mb, *Malus. baccata* Borkh.; Mh, *M. hupehensis* Rehd.; Mm, *M. micromalus* “qingzhoulinqin”; Mr, *M. robusta* Rehd.

Non-enzymatic scavengers and antioxidative enzymes play critical roles in detoxification of ROS in plants exposed to Cd stress, so we examined free proline, soluble phenolics, ascorbate (ASC), total thiols (T-SH), reduced GSH, and sevreal antioxidative enzymes (Figures 5, 6). The species-specific differences in non-enzymatic antioxidants were significant in all tissues except for free proline in wood and ASC in bark (Figure 5). Among the analyzed apple rootstocks, concentrations of free proline were highest in bark of *M. hupehensis* and in leaves of *M. baccata*. Upon Cd exposure, concentrations of free proline were increased in all tissues except in bark of *M. robusta*, compared to control plants (Figure 5). Cd exposure led to significant increases in concentrations of soluble phenolics only in roots of *M. robusta*, while marked increase of soluble phenolics were found in other three tissues of apple rootstocks except in wood of *M. micromalus* “qingzhoulinqin” and *M. robusta*, and in bark of *M. hupehensis* (Figure 5). Cd treatment increased ASC concentrations in all tissues of four apple rootstocks, whereas in resulted in marked decline in wood of *M. baccata* or unchanged in bark of *M. micromalus* “qingzhoulinqin” and in leaves of *M. hupehensis* (Figure 5). Among the four apple rootstocks, the highest concentrations of T-SH and GSH were all found in bark and leaves of *M. baccata*, averaged by 45.9–101.3%, and 62.1–89.2% higher than in those of other rootstocks, respectively. Treatment with 300 μM Cd decreased T-SH and GSH concentrations in tissues of most apple rootstocks in comparison with controls, which was more significant in roots of *M. baccata* (Figure 5).

Changes in antioxidative enzymes of apple rootstocks were also species-specific (Figure 6). Under 300 μM Cd exposure, the activity of SOD was markedly supressed in roots of *M. robusta*, in wood of *M. micromalus* “qingzhoulinqin,” in bark of *M. baccata* and *M. robusta*, and in leaves of *M. micromalus* “qingzhoulinqin” in comparison with those under control conditions (Figure 6). In contrast, SOD activities were significantly enhanced by 48.6% in wood of *M. baccata*, by 17.8 and 21.6% in leaves of *M. baccata* and *M. hupehensis* after the imposition of Cd stress (Figure 6). In most cases, Cd treatment resulted in higher CAT activities in root, wood, and leaf tissues of four apple rootstocks, while repressed activities of CAT in bark of all plants with the lowest reduction found in *M. baccata* (Figure 6). In general, GPX activities were higher in roots and wood, but lower in bark of analyzed apple rootstocks exposed to Cd than those under control conditions. GPX activities were always highest in all tissues of *M. baccata* among four investigated apple rootstocks. Upon Cd exposure, activities of APX were induced in roots of *M. baccata* and in wood of *M. baccata* and *M. micromalus* “qingzhoulinqin,” but inhibited in bark and leaves of most investigated apple rootstocks (Figure 6). In contrast to other antioxidative enzymes, GR activities were repressed in root, wood and bark tissues of four apple rootstocks, but were significantly increased by 80.4 and 69.1% in leaves of *M. baccata* and *M. micromalus* “qingzhoulinqin,” respectively (Figure 6).

## Discussion

### Variation in Cd tolerance and accumulation among different apple rootstocks

Many indicators, such as photosynthesis, chlorophyll, tissue biomass, and tolerance indexs (TI) have been used to evaluate metal toxicity in plants (Shi and Cai, 2009; Zhang et al., 2013). When tissue Cd concentrations are greater than 5–10 ug g^−1^ dry weight, plants will suffer toxic effects (White and Brown, 2010). After 300 μM Cd exposure for 45 days, all seedlings survived which suggests that apple rootstocks are moderately tolerant to Cd stress. However, the negative effects of Cd on photosynthesis, chlorophyll and biomass were found in these rootstocks (Tabels S1, S2). Cd stress caused decline in wood, bark, and leaf biomass, but have no effects on root biomass indicates the aerial parts are more sensitive to Cd than roots. These results are consistent with the finding in eight energy crops (Shi and Cai, 2009). Among four apple rootstocks, the least reduction in plant biomass and the highest TI of the whole plant in *M. baccata* suggest that this apple rootstock is superior to the other three apple rootstocks in terms of Cd tolerance under current experimental conditions.

Root retention, the restriction of metal translocation to the aerial parts, is one of the important mechanisms for metal tolerance in plants (Chaoui et al., 1997; Nada et al., 2007). In the present study, most Cd absorbed in apple rootstocks was retained in roots (Figures 1A, 2A), suggesting that these plants could limit translocation of Cd to the aerial parts, which are in accordance to previous studies (Gill et al., 2012; Jakovljević et al., 2014; Ma et al., 2014). In the aerial parts, Cd was mainly accumulated in bark of apple rootstocks especially in *M. baccata*, likely providing protection to photosynthetic organs of leaves (Lux et al., 2011). It has long been recognized that Cd accumulation ability differs markedly among different species or varieties (Clemens et al., 2013). The tested apple rootstocks in the study displayed different results in Cd concentrations and total amounts (Figures 1, 2A). Among the four apple rootstocks, the lowest Cd concentrations in roots, wood, and leaves, as well as lowest total Cd amounts of *M. baccata* indicate that this apple rootstock has greater ability to avoid Cd absorption and restrict its translocation to the aerial parts, which confers its relative higher Cd tolerance than the other three apple rootstocks. This suggestion is also supported by BCFs results (Figure 2B). Previous studies have demonstrated that metal absorption and transport ability were affected by various edaphic conditions (Jakovljević et al., 2014). However, our previous study also shown that *M. baccata* is superior to the other three apple rootstocks in terms of preventing accumulation of Cd in roots and aerial tissues under low Cd (50 μM) exposure conditions (Zhou et al., 2016), indicating that this apple rootstock may have well-coordinated physiological mechanisms to limit Cd accumulation and translocation, and diminish deleterious effects.

### Cd accumulation and tolerance is mediated by Cd subcellular distribution and chemical forms

The compartmentalization of Cd at subcellular levels is extremely important for Cd accumulation and tolerance in plants (Xin et al., 2013). Cell wall, the first barrier for Cd uptake, is comprised of cellulose, hemicellulose, pectin and protein, and therefore can bind with Cd and restrict it transportation to the cytoplasm (Gallego et al., 2012). In addition to cell wall, vacuole which comprises as much as 90% of the total cell volume in mature plant cell (Pittman, 2005), is rich in sulfur-rich peptides and organic acids, therefore can chelate and compartmentalize Cd in plants (Di Toppi and Gabbrielli, 1999; Clemens et al., 2002). Previous studies have demonstrated that soluble fraction were consisted mostly of vacuoles (Dou et al., 2009; Wang et al., 2016) which acts as the key site of preferential Cd binding in ramie (Wang et al., 2008), barley (Wu et al., 2005), maize (Lozano-Rodriguez et al., 1997), and tomato (Hasan et al., 2015). In accordance with the above results, the vacuoles isolated from plant cells contained most or virtually all Cd present in the protoplast, only little were found in cytoplasm (Ma et al., 2005; Sylwia et al., 2010). Using energy-dispersive x-ray microanalysis microanalysis, He et al. (2013b) also found subcellular Cd compartmentalization occurred mainly in cell wall and vacuole and no Cd was detected in cytoplasm. In spite of this, it is still required to differentiate and analyze Cd compartmentation between the cytoplasma and vacuole in our future research. In the presnet study, the majority of Cd in tissues of four apple rootstocks were stored in cell wall (F I) and soluble fraction (F IV) (most likely in vacuoles), indicating that compartmentalization of Cd in cell wall and soluble fraction is an effective and the most important mechanism for Cd tolerance in all tissues of apple rootstocks. This strategy could further limit Cd transportation from roots to the aerial parts, which were confirmed by lower Cd concentrations and total amounts in the aerial parts of plants (Figures 1, 2).

The subcellular distribution of Cd in different fractions of tissues differed among four apple rootstocks, which may be attributed to the variable levels of Cd tolerance of different plants (Wang et al., 2015a). Compared to the other three apple rootstocks, higher Cd concentrations or distribution ratios of F I were found in *M. baccata*, suggesting that this species may have higher Cd tolerance and adopt more efficient strategy to limit Cd transport. Consistent with the findings by Wu et al. (2005), Cd was mainly distributed in cell wall of Cd-tolerant barley genotype. In plants cells, the decrease in cytosolic concentration of soluble Cd ion is one of the defense strategies against Cd stress, by which way plant cell could avoid Cd accumulation in membrane or the cytosol organelle by immobilizing Cd in subcellular compartments, thus confers enhanced Cd tolerance (Luo et al., 2016). In agreement with the lower reduction in *A* and total biomass, and higher TI of the whole plant, Cd concentrations and distribution ratios in organelle-rich fraction (F II) and membrane-containing fraction (F III) were lower in roots, wood, and leaves of *M. baccata* than those of other apple rootstocks, suggesting this species has relative stronger ability in preventing Cd interfering with the organelles and higher Cd tolerance.

In addition to Cd subcellular distribution, the toxicity degree and mobility of Cd in plants are also dependent on its chemical forms inside cells (Su et al., 2014). Generally, Cd in inorganic and organic forms (extracted by 80% ethanol and deionized water) have higher migration capacity and more toxic to plant cell, compared to pectates and protein integrated forms (extracted by 1 M NaCl), undissolved Cd phosphate forms (extracted by 2% HAC) and cadmium oxalate forms (extracted by 0.6 M HCl) (Wu et al., 2005; Wang et al., 2008). In the present study, it was evident that a majority of Cd was integrated with pectates and protein or phosphate ligands in all tissues of apple rootstocks (Table 2), indicating they have lower capacity to transport Cd, which is considered as an important Cd tolerance mechanism. Consistent with our results, Wang et al. (2015a) also found that to convert Cd into undissolved phosphate forms and pectate/protein-bound forms may be the primary strategy in reducing Cd mobility and toxicity in watercress. Among four apple rootstocks, Cd toxicity in root, wood, and bark tissues was counteracted mainly by integration with peptide ligands, whereas alleviation of Cd toxicity in leaves was primarily through the aggregation by both phosphate and peptide ligands (Table 2). It has been recognized that Cd insoluble phosphate mainly exists in cell wall and vacuole, while pectates/protein-integrated Cd is primarily compartmented in vacuole (Qiu et al., 2011). Therefore, the results of Cd chemical forms in apple rootstocks corresponded well with the results of Cd subcellular distribution. The higher concentrations and proportions of Cd extracted by deionized water than those extracted by 80% ethanol indicate that Cd in apple rootstocks was transported mainly in water soluble forms, which was in line with the findings in *Brassica napus* (Mwamba et al., 2016). The lower concentrations and proportions of water soluble Cd in leaves than those in other tissues may be responsible for the protection of photosynthesis organs from Cd stress. Contrast to the current results, a larger percentages of 80% ethanol-extractable Cd or water soluble organic Cd over non-toxic Cd complexes were reported in roots of *Phytolacca americana* L. (Fu et al., 2011) and *Kandelia obovata* (Weng et al., 2012), these conflicting results may be attributed to the distinct experimental conditions and the variable levels of Cd tolerance in different plants.

In general, much tolerant genotypes or cultivars are endowed with greater ability in reducing free Cd ion inside cells, partly through conversion it into less mobile forms (Hall, 2002). Correspondingly, it is believed that low-Cd accumulation genotypes or cultivars would theoretically have higher proportions of Cd in integrated with pectates and protein forms, insoluble Cd phosphate forms and cadmium oxalic than those with high-Cd accumulation, which are presumably due to lower migration of these forms resulting in lower translocation rate from roots to the aerial parts (Wang et al., 2015a). In the present study, results obtained by sequential extraction technique showed that the variation of Cd concentrations and proportions in different chemical forms existed in four apple rootstocks (Table 2), matching well with distinct level of Cd accumulation and tolerance. Compared to other three apple rootstocks, *M. baccata* had lower concentrations and proportions in mobile forms (inorganic and organic water-soluble Cd) in roots, wood, and leaves, but relative higher percentage of pectate-/protein-bound Cd in roots and wood, phosphate-associated Cd in bark and leaves, and oxalic Cd in all tissues, suggesting that *M. baccata* may adopt more efficient ways to reduce Cd mobility and toxicity and may be superior to the other three apple rootstocks in terms of Cd tolerance.

### Importance of carbohydrate status and antioxidant defense in Cd tolerance

It has been well documented that Cd stress will induce ROS production MDA accumulation (Romero-Puertas et al., 2012; Sandalio et al., 2012). In the present study, the increase of ${\text{O}}_{2}^{•-}$ and H_2_O_2_ production in apple rootstocks after Cd exposure reflected the oxidative stress induced by Cd. However, less pronounced induction of ${\text{O}}_{2}^{•-}$ in roots and bark, and H_2_O_2_ in roots and leaves in *M. baccata* indicate that *M. baccata* may experience less oxidative stress than other three apple rootstocks. Consistent with the ROS results, the accumulation of MDA after Cd exposure was lower in *M. baccata*. These results suggest that *M. baccata* may adopt well-coordinated physiological regulation mechanisms under Cd stress in comparison with other three apple rootstocks.

A comparative analysis of carbohydrate changes and antioxidant defense to Cd stress in plants differing in Cd tolerance could be another way to detect possible tolerance traits (Ma et al., 2014). To counteract Cd induced oxidative stress, total soluble sugars and starch are needed to provide energy. In the present study, four apple rootstocks exhibited large variation in concentrations of soluble sugars and starch, irrespective of the Cd treatments. In agreement with lower accumulation of ROS and MDA, the increases in total soluble sugars in bark, and starch in roots and leaves of *M. baccata* under Cd exposure may contribute to osmotic regulation and oxidant detoxification. Similar results were also found in maize (Anjum et al., 2016) and poplar (He et al., 2013a). In comparison with *M. hupehensis* and *M. robusta*, the constructive higher concentration of starch in roots, bark and leaves of *M. baccata* could be considered as an important Cd tolerance mechanism.

To cope with Cd toxicity, one of the important mechanisms in plants is the induction of non-enzymatic metabolites and antioxidative enzymes (Lin and Aarts, 2012). The significant species-specific differences in antioxidants among four apple rootstocks suggest that different stress defense pathways exist in these plants. It has been well documented that free proline, soluble phenolics, ASC, T-SH, and GSH play a key role in the alleviation of Cd toxicity by detoxifying ROS (Xu et al., 2009; Chen et al., 2011; He et al., 2015). The stimulation of free proline, soluble phenolics and ASC in tissues of most analyzed apple rootstocks may play a role in Cd detoxification under Cd stress, as observed in other plants (Wu et al., 2004; Podazza et al., 2012; Xu et al., 2012a). A higher accumulation of these three compounds in *M. baccata* supports the observed higher Cd tolerance in *M. baccata* than other three apple rootstocks, corresponding well to lower accumulation of ROS and MDA. As both a component of the GSH-ASC cycle and metal chelator, GSH not only participate in the removal of excess H_2_O_2_, but also can chelate directly with metal, or be utilized as a precursor for phytochelatins (PCs) biosynthesis to decrease metal toxicity in plant cells often becomes depleted after metal exposure (Szalai et al., 2009; Seth et al., 2012). In roots of apple rootsocks, lower GSH concentrations were detected after Cd stress, especially in *M. baccata*, which was probably due to the formation of Cd-GSH complexes or the biosynthesis of PCs. Moreover, T-SH and GSH concentrations in bark and leaves of *M. baccata* were always higher than those in tissues of other apple rootstocks, irrespective of Cd treatments, indicating that this apple rootstock may have strong abilities to scavenge ROS or chelate with Cd.

In addition to non-enzymatic metabolites, antioxidative enzymes such as SOD, CAT, GPX, APX, and GR are also of great importance to overcome Cd induced oxidative stress injury (Chen et al., 2011; Nahar et al., 2016; Podazza et al., 2016). In agreement, the present study found elevated activities of SOD, CAT, GPX, and APX in roots and wood, and SOD, CAT, and GR in leaves of *M. baccata* after Cd treatment. Higher antioxidative enzyme activities suggest that *M. baccata* has a higher free radical-scavenging capacities corroborated by lower ROS concentrations and lipid peroxidation, which may result in higher Cd tolerance. In contrast to other antioxidative enzymes, the remarkably supressed of GR activities by Cd in roots, wood, and bark of apple rootstocks was found and probably due to inactivation, because Cd can combine with the sulphydryl groups of GR (Van Assche and Clijsters, 1990), as documented in *Populus yunnanensis* (Chen et al., 2011).

In conclusion, the four apple rootstocks were moderately tolerant to high Cd exposure and variations in Cd tolerance existed among these rootstocks. Cd exposure caused negative effects on photosynthesis, chlorophyll and biomass in four apple rootstocks, which was less pronounced in *M. baccata*. In comparison with the other three apple rootstocks, *M. baccata* displayed the lowest Cd concentrations in roots, wood and leaves, the smallest total Cd amounts as well as the lowest BCF, indicating that this apple rootstocks may has greater ability to avoid Cd absorption and restrict its translocation to the aboveground parts which may contribute to its relative higher Cd tolerance. Cd subcellular distribution and chemical forms are relevant to species differences in Cd tolerance of four apple rootstocks. The depositions of Cd in the cell walls and soluble fraction (most likely in vacuoles) and the formations of precipitates with pectate/protein or phosphate ligands were the key strategies associated with the Cd tolerance and detoxification in apple rootstocks. Among these four apple rootstocks, *M. baccata* had lower concentrations of ${\text{O}}_{2}^{•-}$ in roots and bark, H_2_O_2_ in roots and leaves and MDA in roots, wood, and bark; higher concentrations of soluble sugars in bark and starch in roots and leaves; elevated concentrations of free proline, soluble phenolics and ASC; higher concentrations of total thiols (T-SH) and GSH in bark and leaves; and enhanced activities of SOD, CAT, GPX, and APX in roots and wood, and SOD, CAT, and GR in leaves after Cd exposure. These data indicate that the relative higher Cd tolerance in *M. baccata* than other three apple rootstocks under the current experimental conditions is mainly ascribed to Cd subcellular distribution and chemical forms as well as well-coordinated physiological regulation mechanisms. These results will be conducive to the selection of Cd-tolerant apple rootstocks with low Cd accumulation.

## Author contributions

JZ, HW, JH, and DL conceived and designed research. JZ and HW conduced the laboratory works. JZ, JH, and DL analyzed data and prepared the manuscript. HL provided plant materials. All authors read and approved the manuscript.

### Conflict of interest statement

The authors declare that the research was conducted in the absence of any commercial or financial relationships that could be construed as a potential conflict of interest.
